# A new trophic specialization buffers a top predator against climate-driven resource instability

**DOI:** 10.1093/beheco/arae005

**Published:** 2024-01-17

**Authors:** Laura Gangoso, Duarte S Viana, Marina Merchán, Jordi Figuerola

**Affiliations:** Department of Biodiversity, Ecology and Evolution, Faculty of Biological Sciences, Complutense University of Madrid, C/Antonio Novais 12, 28040, Madrid, Spain; Estación Biológica de Doñana, CSIC, C/Américo Vespucio 26, 41092, Sevilla, Spain; Estación Biológica de Doñana, CSIC, C/Américo Vespucio 26, 41092, Sevilla, Spain; Estación Biológica de Doñana, CSIC, C/Américo Vespucio 26, 41092, Sevilla, Spain

**Keywords:** breeding success, foraging behavior, genetic color polymorphism, phenotypic plasticity, population dynamics, resource selection

## Abstract

Intraspecific phenotypic variability is key to respond to environmental changes and anomalies. However, documenting the emergence of behavioral diversification in natural populations has remained elusive due to the difficulty of observing such a phenomenon at the right time and place. Here, we investigated how the emergence of a new trophic strategy in a population subjected to high fluctuations in the availability of its main trophic resource (migrating songbirds) affected the breeding performance, population structure, and population fitness of a specialized color polymorphic predator, the Eleonora’s falcon from the Canary Islands. Using long-term data (2007–2022), we found that the exploitation of an alternative prey (a local petrel species) was associated with the growth of a previously residual falcon colony. Pairs in this colony laid earlier and raised more fledglings than in the other established colonies. The specialization on petrels increased over time, independently of annual fluctuations in prey availability. Importantly, however, the positive effect of petrel consumption on productivity was stronger in years with lower food availability. This trophic diversification was further associated with the genetically determined color morph, with dark individuals preying more frequently on petrels than pale ones, which might promote the long-term maintenance of genotypic and phenotypic diversity. We empirically demonstrate how the emergence of an alternative trophic strategy can buffer populations against harsh environmental fluctuations by stabilizing their productivity.

## Teaser

Organisms often have different strategies to deal with fluctuations or changes in their environment. Elenora’s falcons typically specialize on migrating birds that are blown to nesting sites on the prevailing winds. These food sources are highly ephemeral and unpredictable leading to variation between years in breeding success. In one population of falcons, some individuals, mostly of a particular color morph, have switched to a new foraging strategy that targets locally abundant seabirds. This switch has allowed falcons in that population to cope when their traditional food sources are scarce promoting population persistence and expansion.

## INTRODUCTION

Generalist and specialist strategies often coexist in nature, between and within species and populations, but their relative success clearly depends on the environmental context and its stability. In general, specialists are more sensitive to environmental changes than generalists (Clavel et al. 2011). Indeed, specialist species are declining faster and are predicted to have a higher extinction risk under contemporary environmental change than generalist species worldwide (Colles et al. 2009). For example, some species specialized in exploiting pulse resources are facing phenological mismatches due to global warming (Visser and Both 2005). These negative consequences may, however, be counteracted by intraspecific behavioral plasticity along a continuous gradient of specialization, enhancing the ability of populations and species to cope with environmental variation and changes (Piersma and Drent 2003; Reusch et al. 2005; Bell and Gonzalez 2009; González-Suárez and Revilla 2013).

Phenotypic diversification can occur among individuals, or the same individual can switch between different strategies (i.e., reversible state effects, Senner et al. 2015), such as phenological (e.g., facultative partial migration in birds, Jacobs and Wingfield 2000; Boyle 2008) and foraging strategies (e.g., Tinker et al. 2008). Individual diet specialization, which occurs when some individuals exploit preferentially a subset of prey types included in the trophic niche of the whole population (Bolnick et al. 2002, 2003), may be driven by different processes such as intra- and interspecific competition, ecological opportunity, and predation (Araújo et al. 2011; Costa-Pereira et al. 2018). For example, high population densities may increase the levels of intraspecific competition, which may, in turn, promote rapid diet diversification among co-occurring individuals, depending in many cases on their phenotypes (Svanbäck and Bolnick 2007; Huss et al. 2008). In addition, resource diversity per se may promote opportunistic trophic niche diversification within a population (Darimont et al. 2009; Semmens et al. 2009). In any case, the emergence of trophic specialization is more likely when individuals differ in their ability to detect, capture, or handle alternative food resources (Boag and Grant 1981).

Here, we documented the emergence of a new trophic strategy over a period of 16 years in a population subjected to strong fluctuations in the availability of its main trophic resource and assessed how this diet diversification affected the breeding performance and population fitness. We addressed this question using a color polymorphic bird predator, the Eleonora’s falcon (*Falco eleonorae*), which is highly specialized in migratory songbirds as a food resource during the breeding season. As a consequence of this specialization, both the timing of breeding (mid-July to October) and the location of breeding colonies on islands across the Mediterranean basin are finely adjusted to intercepting songbird migratory routes. Although the migration flux is typically regular across the Mediterranean, in the western-southern edge of the species’ breeding range—the Canary Islands—this flux is more unstable, depending strongly on the intensity of Atlantic trade winds (Gangoso et al. 2020). This instability causes annual variation in the population’s productivity, including extremes of complete breeding failure (no fledglings survive, as already observed; Gangoso et al. 2020). In fact, productivity in this population shows higher fluctuations than in any other population of the species (e.g., Xirouchakis et al. 2012).

Eleonora’s falcons of both sexes display a genetically determined and heritable melanin-based color polymorphism with two discrete morphs derived from a recessive allele (pale) and a dominant allele (dark eumelanic) of the *MC1R* gene (Gangoso et al. 2011). Both morphs co-occur at temporally and geographically stable frequencies (Walter 1979; Ristow et al. 1998), but the frequency of the dark morph is lower than that of the pale one (dark ca. 30% and pale ca. 70%). On the Canary Islands, social dominance relationships cause spatial segregation of nests of males with different morphs (Gangoso et al. 2015), although mating is random with respect to plumage coloration (Gangoso and Figuerola 2019). This segregation has led to different breeding strategies. For example, while pale males are highly colonial and occupy densely populated areas, dark males often defend larger territories located in the periphery of colonies. Dark males (regardless of the female color), however, achieve higher breeding success than pale males in some years despite having similar clutch sizes (Gangoso and Figuerola 2019).

During the long-term monitoring of the Canarian population, we observed a progressive increase in the number of breeding pairs in areas (or colonies) where the number of local seabird species (i.e., petrels) hunted by falcons was apparently increasing, even though petrels had been reported only as anecdotal prey items in the Canarian population (De León et al. 2007) and never in other Mediterranean colonies (Xirouchakis et al. 2019). We hypothesized that this alternative prey could compensate for the highly fluctuating availability of migratory songbirds during the breeding season and explain the higher productivity in colonies where dark males are relatively more abundant (Gangoso et al. 2015). More specifically, we hypothesized that 1) the consumption of local seabirds depends on the male morph and colony, 2) the consumption of seabirds advances the egg-laying date, as migratory birds are still scarce in the beginning of the breeding season (end of July - beginning of August), and 3) productivity increases with the amount of petrels consumed depending on overall food availability, compensating for food instability and stabilizing annual productivity. We also expected that 4) the exploitation of the alternative resource could have led to a concomitant increase of colony size over time due to an increase in productivity.

## METHODS

**Figure 1:**
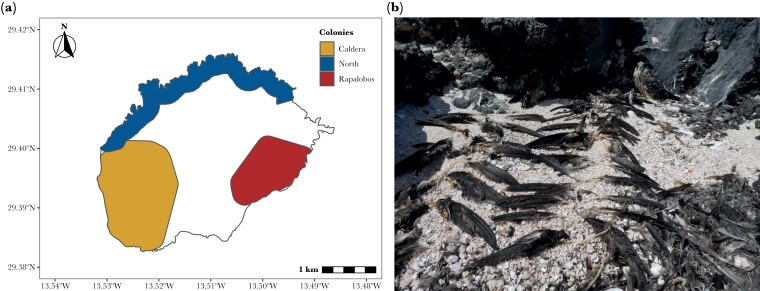
(a) Map of the Alegranza island showing the location (colored polygons) of the different breeding colonies of Eleonora’s falcons. (b) Petrel remains used to quantify the number of preys consumed by the parents and offspring in one nest, where a fledgling can also be seen.

The study was carried out on Alegranza (29°24ʹ N, 13°30ʹ W, 10.2 km^2^, 289 m a.s.l.), one of the small islands in the Chinijo Archipelago, and the northernmost island of the Canary Archipelago. This volcanic island hosts most of the Canarian population of Eleonora’s falcon. Nests are located in different areas within the island, at different altitudes, and with variable conspecific density. In addition, the relative morph frequency of male falcons differs between colonies, with higher frequencies of dark males found in the North as compared with other colonies (Gangoso et al. 2015). We defined three different colonies clearly separated within the island (Figure 1a), where the falcons consistently return each year for breeding: 1) “North,” a basaltic and mostly flat area located at the north side of the island, 2) “Caldera,” a big volcano of about 240 m depth, c.a. 1200 m diameter and an altitude of 289 m, which is situated in the westernmost part of the island and occupies a third of its total area, and 3) “Rapalobos,” which encloses two nearby medium-sized craters (i.e., Rapadura and Lobos), with a maximum altitude of 226 m, which are located East to the Caldera (Gangoso et al. 2015). No nest sites are located on the central part of the island, which consists mainly of lava grounds (i.e., badlands).

During the breeding season, Eleonora’s Falcons feed on migratory birds that are pushed off course by easterly winds, and their availability greatly varies within and across years depending on regional trade winds (Viana et al. 2016; Gangoso et al. 2020). Different petrel species also breed in Alegranza, mainly on the north part of the island, and their breeding season largely overlaps with that of Eleonora’s falcons (Rodríguez et al. 2003). Of these, Bulwers’s petrels (*Bulweria bulwerii*, ca. 150–200 breeding pairs, Rodríguez et al. 2003) and European storm petrels (*Hydrobates pelagicus pelagicus*, ca. 200–300 breeding pairs), whose body sizes are below 100 g (Dunning 2008), have been found in the diet of Eleonora’s falcons (De León et al. 2007). Food provisioning during breeding is carried out by male falcons (Walter 1979).

### Field monitoring

The Eleonora’s falcon population on Alegranza consists on average of 128 breeding pairs (range = 110–132 pairs), which were intensively monitored during July–October 2007–2022. During the study period, a total of 2626 Eleonora’s falcons were ringed with conventional metal rings. Of these, 2243 individuals were also ringed with colored plastic rings that allow for individual identification at a distance. Every year and for each nest site, we recorded the morph of both parents using a spotting scope (*N* = 1330 nesting events). The reliability of our visual morph scoring was confirmed molecularly (Gangoso et al. 2011). We visited nests (range = 73–113 nests/year) on at least two occasions each year, first to quantify the clutch size and record the geographic position on a GPS, and posteriorly to determine the final number and color morph of fledged offspring. For analyses assessing variation of laying date, we considered only nests where reproduction was initiated, where chicks survived long enough as to calculate their age (see below), which reduced the sample size to 1140 nesting events. The laying date (where 1 = July 1^st^) was calculated by subtracting 30 days (the duration of the incubation period, Wink and Ristow 2000) to the hatching date of the older chick within a brood, which was calculated after measuring nestling wing length to estimate their age (± 1 day) using the formula provided by Ristow and Wink (2004). The number of fledglings was estimated as the final number of fledged offspring from each nest. The mean annual productivity of the population was estimated as the number of fledglings divided by the number of breeding pairs monitored.

We quantified the prey in each nest visited in two ways. As a general phenomenon, Eleonora’s falcons store the leftover prey in larders located in caves and bushes around the nest site (Viana et al. 2016; Gangoso et al. 2020). We recorded the presence of prey and prey remains (feathers and body parts, mostly passerines) within a 5-m radius of the nest site by inspecting the nest and nearby larders. Because falcons consume most of the prey, leaving only scattered remains that are difficult to quantify, we focused on petrel remains, which are easily quantified by the number of intact wing pairs that falcons do not consume (Figure 1b). This method allowed us to identify prey species (according mostly to feathers) and to quantify the number of petrels in the diet. In addition, in 2017 and 2018, we installed trap-cameras (Moultrie digital game camera, model M-40i) in 40 and 18 nests, respectively, to quantify the number of preys delivered daily to offspring during the chick-rearing period, that is, from recently hatched to approximately 24 days old chicks. Eleonora’s falcons hunt several bird species of different sizes, from small passerines (the vast majority) to larger-sized preys, such as European turtle doves (*Streptopelia turtur*), European quails (*Coturnix coturnix*) and some petrel species (De León et al. 2007). Because female falcons usually pluck prey and dispose of inedible parts (i.e., wings, legs, etc.) before entering the nest to feed nestlings, we could quantify the total number of preys consumed but not precisely identify the species delivered in most cases. Cameras recorded a total of 963,465 pictures. Photographs were inspected using FastStone Image Viewer (version 7.4). In some cases, it was not possible to see the exact number of preys that were delivered due to technical failures of the cameras (running out of battery, camera dropped because of the falcons, wrong placement of the camera). Therefore, these 534 incomplete days were excluded from the analysis.

Food availability during September (the chick-rearing period in Eleonora’s falcons) was estimated for every study year in two ways. 1) The intensity of easterly winds across the migratory corridor that connects Europe (southwest Iberian Peninsula) and the Canary Islands has been proven to control the number of migratory birds arriving to Alegranza during the falcons’ breeding season, explaining 70% of the variation in annual productivity (Gangoso et al. 2020). We calculated the mean easterly wind intensity as a proxy of overall food availability (see Gangoso et al. 2020 for details). However, this is a proxy that does not capture other important aspects that control prey availability, such as the intensity of the migratory flux between Europe and Africa. 2) The ultimate measure of annual prey availability is the mean annual productivity, which depends almost entirely on prey availability due to the lack of predators or any other limiting environmental factors. Therefore, we used the mean annual productivity as a proxy of overall food availability.

Permits for field work were granted by Spanish Regional (Gobierno de Canarias) and Local (Cabildo de Lanzarote) Administrations. Specific permissions numbers: MAOT N° 11908, MAOT N° 6468, MAOT N° 9723, MAOT N° 9498, Res: 2255, 2273, 2129, 1908, 3301, 1876, 2642, 2272, 1453, 1799, 10521, 349/2014, 343/2015, 278/2017, 61/2018, 3395/2019, 110/2020, 4447/2021, 4285/2022.

### Statistical analyses

To test the first hypothesis (i.e., that the consumption of petrels depends on the male morph and the location of the nest site), we assessed whether the number of petrels consumed by male falcons throughout the breeding period was associated with their color morph and the colony by fitting a generalized linear mixed model (GLMM) with a negative binomial error distribution and log-link function with the package *lm4* (Bates et al. 2015). In this model, the interaction between male morph and colony was also considered, and the year was included as a random intercept. We used a negative binomial distribution to account for overdispersion observed using a Poisson GLMM. We used a similar GLMM to assess the relationship between the male morph and the number of petrels hunted using only nests within the North colony. In addition, we assessed whether the number of all prey items delivered to nestlings throughout the nestlings’ development period was associated with the male color morph and the colony, while accounting for food availability and demand. To this end, we used a GLMM with Poisson error distribution, with the number of nestlings present in each nest each day, their age, and the daily availability of food as independent continuous variables, and nest as a random intercept. Daily food availability was estimated according to the wind patterns registered daily between August 17th and September 26th (the trap-cameras monitoring period) in 2017 and 2018. In all models, covariates were centered by subtracting the mean of all data points from each individual data point. Fixed effects in the GLMM models were tested using likelihood ratio tests (see Supplementary Tables S1–S3 for detailed statistical information on each model).

Then, we assessed whether the exploitation of an alternative food resource (i.e., petrels) was associated with variation in the laying date (hypothesis 2), by fitting a LMM where the laying date was the response variable and the number of petrels in the nest was the only predictor, while allowing for randomly varying intercepts per year. The hypothesis 3 that the number of fledglings raised was related to the number of petrels consumed and/or laying date, and the annual food availability was tested with cumulative link mixed models (CLMM) fitted with the Laplace approximation, using the *clmm* function of the R package *ordinal* (Christensen 2018). The response variable was the ordered productivity (4>3>2>1>0), the number of petrels in the diet, overall prey availability (either mean easterly wind intensity or annual productivity), and their interaction were the explanatory variables, and the year was included as a random intercept. The interaction assessed whether the effect of petrels on per nest reproductive success depended on the availability of food during the breeding season. To assess the mean annual effect of this interaction and test for a buffering effect of petrel consumption on annual productivity, we tested whether petrel consumers raised more offspring than non-consumers depending on food availability. We modeled the annual difference in the mean number of fledglings raised by petrel consumers versus non-consumers as a function of either annual prey availability or mean annual productivity using linear regression. In these models, both prey availability and mean annual productivity were included as the only explanatory variables, respectively. In addition to the effect of petrel consumption and food availability, we also assessed whether the number of fledglings raised was associated with the laying date by fitting a CLMM where the laying date was the only explanatory variable, while allowing for randomly varying intercepts per year. We used CLMMs as an alternative to GLMMs with a Poisson error distribution because the model errors in the latter were underdispersed. Fixed effects in the CLMM models were tested using likelihood ratio tests (see details in Supplementary Tables S3–S5).

Lastly, we tested the hypothesis (4) that the number of breeding pairs (as assessed by the number of nests) in the different colonies changed throughout the years. The mean number of petrels per year was calculated as the number of petrels divided by the number of nests sampled each year across the entire island. We assessed whether the mean number of petrels hunted changed over time by fitting a LM with year as the only fixed factor. We modeled the number of nests (log-transformed) as a function of the mean number of petrels per year, the colony, plus their interaction using a linear model. In addition, we assessed whether the mean number of nests changed across years by fitting a LM with year, colony, plus their interaction as fixed factors. Analyses were performed in R software (v. 4.0.0; R Core Team 2019). R code used is available in Supplementary File 1.

## RESULTS

**Figure 2:**
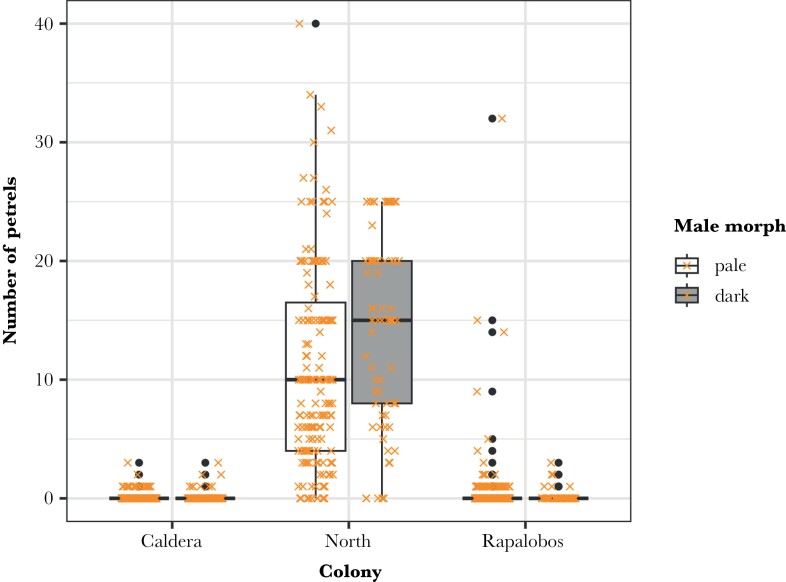
Boxplot showing the number of petrels consumed in the sampled nests of each colony, distinguishing the counts by the male’s morph. The lines within the boxes indicate the median, the edges of the boxes the first (Q1) and third (Q3) quartiles, and the whiskers extend to 1.5 times the interquartile range. Dots represent extreme values outside the boxplot’s whiskers, and the crosses are the data.

We found petrel remains in 322 of the 1330 nests monitored across all the study years. These nests were distributed across the whole island; however, the number of petrels captured was clearly higher in the North (remains found in 223 nests, estimate ± standard error = 5.27 ± 0.23 SE) than in the other two colonies (32 nests in Caldera and 67 in Rapalobos, est. = 1.60 ± 0.23 SE, LR test χ^2^ = 847.22, df = 2, *P* < 0.001) (Figure 2). Dark males hunted significantly more petrels than pale ones (est. = 0.73 ± 0.36 SE, LR test χ^2^ = 5.34, df = 1, *P* = 0.02). The interaction between these two variables was also significant (LR test χ^2^ = 7.19, df = 2, *P* = 0.03) and showed that dark males breeding in the North and in the Caldera colonies hunted more petrels than pale ones compared to the Rapalobos colony (Figure 2, Supplementary Table S1). This difference between morphs was held when considering only falcons breeding in the North colony (est. = 0.27 ± 0.11 SE, LR test χ^2^ = 6.12, df = 1, *P* = 0.01, Supplementary Table S2). On the other hand, the overall number of preys (i.e., migrating birds, independently of their identity) hunted and delivered to nestlings each day depended only on the number of nestlings present (est. = 0.14 ± 0.03 SE, LR test χ^2^ = 16.98, df = 1, *P* < 0.001) and their availability (est. = −0.03 ± 0.005 SE, LR test χ^2^ = 40.43, df = 1, *P* < 0.001, Supplementary Table S3).

**Figure 3:**
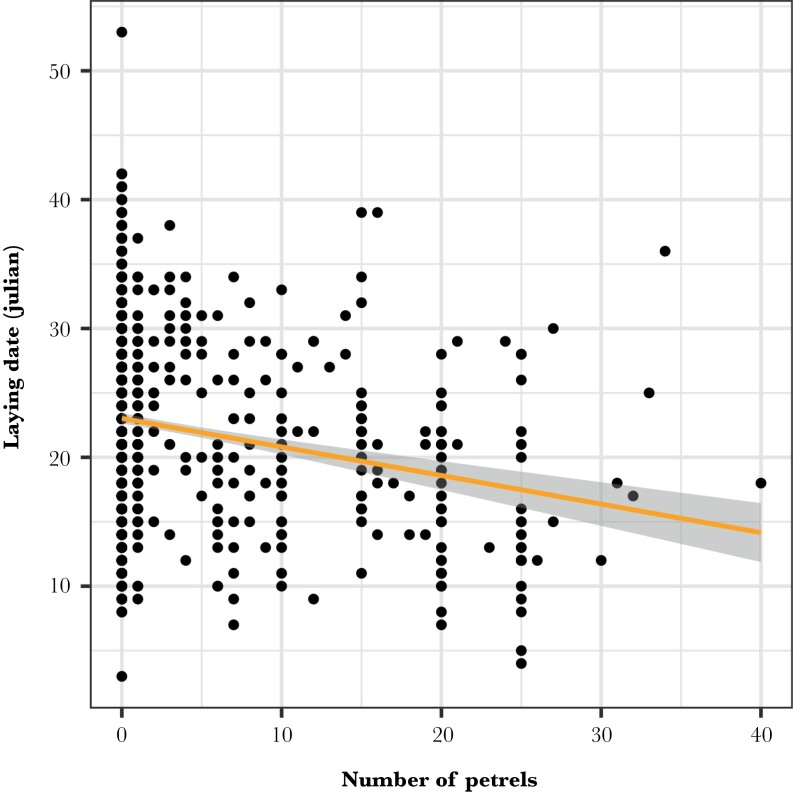
Association between laying date and the number of petrels consumed. The line represents the linear model with the respective 95% CI gray bands.

**Figure 4:**
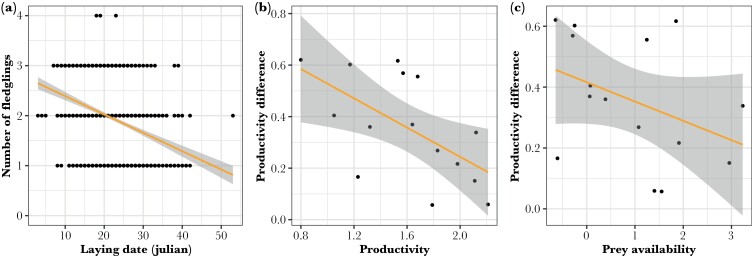
Correlates of reproductive success are measured as the number of fledged offspring. (a) Association between the number of fledged offspring per nest and laying date. (b, c) Difference between the annual mean number of fledglings raised by petrel consumers versus non-consumers (productivity difference) as a function of either the mean annual productivity across all nests (b) or the overall availability of prey as measured by the annual mean intensity of easterly wind.

The laying date was strongly associated with the number of consumed petrels (est. = −0.26 ± 0.03 SE, LR test χ^2^ = 74.87, df = 1, *P* < 0.001): the higher the number of petrels, the earlier the laying date was (Figure 3, Supplementary Table S4). The number of fledglings raised was positively related to prey availability (est. = 0.57 ± 0.13 SE, LR test χ^2^ = 11.01, df = 1, *P* < 0.001) and the number of petrels (est. = 0.07 ± 0.01 SE, LR test χ^2^ = 48.86, df = 1, *P* < 0.001), but the effect of petrel consumption on reproductive success decreased with increasing prey availability (est. = −0.01 ± 0.01 SE, LR test χ^2^ = 3.02, df = 1, *P* < 0.08), especially when using mean annual productivity as a proxy of net prey availability (est. = −0.05 ± 0.02 SE, LR test χ^2^ = 4.60, df = 1, *P* = 0.03) (Supplementary Table S5). The number of fledglings raised was also negatively related to the laying date (est. = −0.09 ± 0.01 SE, LR test χ^2^ = 101.18, df = 1, *P* < 0.001) (Figure 4a).

The difference in mean number of fledglings raised between nests with and without petrels was higher in years with lower prey availability (Figure 4b,c). This trend was statistically significant when using annual productivity (est. = −0.28 ± 0.11 SE, *P* = 0.02, adjusted *R*^2^ = 0.30, Figure 4b), but not the wind proxy (est. = −0.06 ± 0.04 SE, *P* = 0.15, Figure 4c), indicating that petrel consumption compensates the low overall productivity in years with low prey availability.

**Figure 5:**
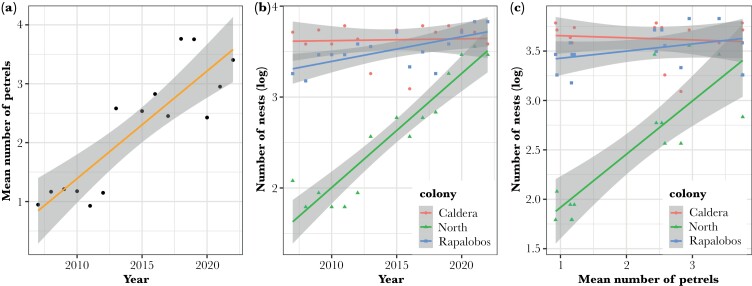
The rise of the North was associated with the number of petrels consumed. (a) The mean number of consumed petrels across nests in the whole island throughout the study period. (b) Log-number of nests throughout the study period in each colony. (c) The mean number of nests in each colony as a function of the mean number of consumed petrels each year (across the whole island).

Overall, the mean number of petrels hunted increased over the study years (year effect: *F*_1,13_ = 39.8, *P* < 0.001, Figure 5a, Supplementary Table S6), and this increase was not related to prey availability estimated from wind patterns (Spearman ρ = −0.007, *P* = 0.81). The mean number of nests remained similar across years in the Caldera and Rapalobos colonies but increased in the North (from only seven nests in 2007 to 32 in 2022; interaction effect: *F*_2,39_ = 35.88, *P* < 0.001, Figure 5b, Supplementary Table S6). Such an increase was strongly associated with the mean number of petrels hunted (interaction effect: *F*_2,39_ = 19.37, *P* < 0.001, Figure 5c, Supplementary Table S6).

## DISCUSSION

Our study is a striking example of how intraspecific trophic diversification can occur fast and sympatrically to buffer the population against climate-driven resource instability, increasing population resilience and fitness. The exploitation of an alternative food resource that does not depend on climatic conditions, particularly in the North colony, led to earlier laying dates and a higher number of fledglings raised. Importantly, the positive effect of petrel consumption on offspring production was stronger in years with lower food availability and consequently lower overall productivity, regardless of the male color morph, showing how this trophic innovation can effectively buffer the population against resource instability and increase population fitness.

The growth of the North colony was accompanied by some changes in the population structure. The frequency of the dark morph was higher in the North colony (Gangoso et al. 2015), and we observed that dark males hunted more petrels than pale ones (although both dark and pale males that consumed petrels showed, on average, higher productivity). Although we could not establish a causal relationship, this result might indicate that this trophic diversification was not a random process, but the consequence of genetic differences that might somehow promote the exploitation of this alternative resource. Amongst other factors, genetic color polymorphism has been often associated with different foraging strategies. Different color morphs usually differ in a range of life-history traits that allow the exploitation of alternative ecological niches (Delhey and Peters 2013), influencing morph-specific predator–prey relationships, foraging success, and therefore, the opportunities for diet specialization (e.g., Roulin 2004; Tate and Amar 2017; Karell et al. 2021).

One possibility is that morph-dependent hunting success depends on the prey species (Galeotti et al. 2003), as found in other birds like barn owls (Roulin 2004) and black sparrowhawks (*Accipiter melanoleucus*) (Tate and Amar 2017). Some studies with the latter species proposed that light conditions could have an influence on hunting success between different morphs, with pale morphs having an advantage under bright conditions and dark morphs under darker conditions (Tate and Amar 2017; Tate et al. 2017). This could be the case of Eleonora’s falcon males if the dark color confers an advantage for hunting seabirds, especially under darker conditions (being less detectable than pale males). Bulwer´s and storm petrels leave and return to their nest sites at dawn and dusk, respectively, and forage over the sea at sunset (Neves et al. 2011), so they could be less able to detect a dark predator under such light conditions. However, not all dark males prey on petrels, and many pale males do. Diet variation can emerge independently of the morph, as observed in other polymorphic species (Collins et al. 1993; Maret and Collins 1997). The relationship between male color morph and the amount of petrels consumed could have originated through spatial segregation of morphs because dark males usually breed in peripheral areas (e.g., due to social dominance relationships; Gangoso et al. 2015). In the beginning of the study period, the north colony was a residual area mainly used by a few breeding pairs mostly composed by dark males, and this area happens to be where petrels are more abundant. Still, both dark and pale falcons coexist in the north and both can hunt petrels, suggesting that the selective consumption of petrels is more linked to the location of the nest site than to the color morph. In addition, we found a high number of petrels in a few nests located in the Rapalobos colony, and these nests were held by pale males. These nests are all located in peripheral areas, and it is highly likely that petrels also breed in high numbers in those same places. In any way, the combined effects of genetic variation and/or plasticity are allowing for trophic niche broadening at the population level.

Living in the north colony and hunting petrels was also associated with changes in laying date, which is remarkable for a species whose breeding phenology is totally synchronized to the timing of the seasonal resource pulse, that is the flux of migratory passerines heading to Africa (Walter 1979). This is not associated with the female’s age or experience, as data on ring recoveries indicate that the distribution of female ages did not differ across the island and that recruitment of young females is equally frequent in all colonies (authors’ unpublished data). Rather, our results suggest that falcons are able to exploit an alternative food resource that does not depend on wind patterns and adjust their breeding time accordingly. Indeed, the increase in the number of petrels hunted was independent of prey availability during September, and observed changes in laying dates were unrelated to the annual availability of the main preys. In general, there is strong selection for early breeding in birds—earlier breeders often produce larger clutches and raise more offspring with higher survival and recruitment rates (Newton and Marquiss 1984; Öberg et al. 2014; Catry et al. 2017). In the Eleonora’s falcon, earlier laying dates are also strongly associated with higher productivity in this and other populations of the species (see also Hadjikyriakou et al. 2020). Although the laying date may depend on factors such as age and experience of both adults (Wink et al. 1993; Forslund and Pärt 1995), egg production is an energetically demanding process, and hence egg laying and clutch size strongly depend on the females’ body condition (Rowe et al. 1994; Descamps et al. 2011). In many species, the female’s condition depends on male feeding behavior during courtship (Catry et al. 2017). In our study area, breeding petrels are abundant between mid-June and August, when reliefs during incubation and chick feeding are more frequent, and immature individuals prospecting breeding areas are also around (Nunes and Vicente 1998; Bradley et al. 1999). Indeed, petrel carcasses are already found around falcons’ nests by mid-July (authors’ personal observation). Therefore, females feeding on this extra resource before the migratory flux peaks may reach an optimal body condition earlier than those relying exclusively on migratory passerines.

The consistently higher than average breeding success of the North colony could also promote the settlement of new recruits through the use of integrated direct and indirect cues (i.e., direct assessment and public information) collected in the present and previous year (Doliguez et al. 2003; Pärt and Doliguez 2003), as found in other facultatively colonial raptors such as black kites (*Milvus migrans*) (Sergio and Penteriani 2005). The high philopatry observed in this species can also contribute to the sustained growth of the colony. In our study area, most male recruits in the north were born there (63.26%, *N* = 49), and in 48.98% of the cases (*N* = 8 pale and 16 dark males), the color morph of the father was dark. In general, the degree of philopatry in birds of prey increases when resources are more predictable and heterogeneously distributed (Newton 1979), and thus, the expansion of the colony may be further facilitated by the high predictability of the new prey.

If this trophic innovation is mainly driven by opportunity, it could be expected that it remains in the North colony, where interactions between falcons and petrels are higher. However, given the higher fitness of this alternative foraging strategy, it is possible that it spreads to the entire population, for example, through cultural transmission when individuals from the North disperse to other areas, and even replace the common strategy until an evolutionarily stable strategy is reached (e.g., Křivan 2010 and references therein). Moreover, the number of petrels seems to have increased in recent years (as suggested by the increasing numbers of fledglings grounded by light pollution recovered each year on Lanzarote; Cabildo de Lanzarote’s own data), which might contribute to further expand the exploitation of the novel resource. So far, this alternative foraging strategy has not spread across the entire island. We do not know the reason, but the fact that the north of the island is most exposed to the open sea and has a higher number of petrel burrows might explain the higher number of preyed petrels in this colony in comparison with the other colonies. It could be the case that it is spreading very slowly, as we have also observed a continuous increase in the consumption of seabirds in other peripheral areas of the island, but only the future will tell.

If the (now) alternative strategy does not spread to the entire island, the foraging diversification may increase variation in individual fitness within the population. Other studies also reported individual differences in diet, such as that of Golet et al. (2000), who found that foraging differences in pigeon guillemots (*Cepphus columba*) were associated with variation in reproductive success—individuals selecting bigger prey for their chicks had to forage and visit their nests less frequently, which reduced the exposure of nestlings to predators and led to higher breeding success. Ultimately, individual behavioral differences can lead to evolutionary change and even alter eco-evolutionary dynamics (Hendry 2016). If the success of the alternative foraging strategy is maintained in this falcon population in the long term, as seems to be the case in recent years, it may cause divergent selection. For example, population growth and climatic changes initiated trade-offs in barnacle geese that led to the development of new life-history strategies (Eichhorn et al. 2009) and to subsequent genetic divergence among breeding areas, suggesting that behavioral changes may drive rapid evolution (Jonker et al. 2013). The emergence of new foraging and breeding strategies in this Eleonora’s falcon population may ultimately promote advances in the timing of breeding and the maintenance of color polymorphism in this species through trophic selection if the strategy remains associated with the morph. However, this advantage cannot completely offset the lack of food, as in fact occurred in 2014, when a prolonged food gap associated with unusual wind conditions during September led to the collapse of the productivity of the entire population (Gangoso et al. 2020), including pairs that consumed petrels.

## CONCLUDING REMARKS

The establishment of the Canarian population of Eleonora’s falcons occurred about a century ago (Polatzek 1908; Bannerman 1963; Bannerman and Bannerman 1965). As such, we are still witnessing the consolidation of this breeding population and how this species managed to expand its range westward. Its natural behavior of hunting migratory birds is still pervasive in the Canarian population, but this resource in the Canary Islands is highly dependent on annual trade winds (Gangoso et al. 2020). We have recorded how the productivity was greatly reduced, and even collapsed, in years with altered wind patterns. At the same time, we have observed how a growing local colony was somehow escaping the harshness of these climatic fluctuations, something that we can now associate with the emergence of a new trophic specialization. Our study shows how individual specialization associated with phenotypic variation of either genetic or plastic origin may affect demographic and life-history parameters, likely affecting phenotypic trait and genotype distributions, and in turn, population dynamics (Hairston et al. 2005; Coulson et al. 2011; Schoener 2011).

## Supplementary Material

arae005_suppl_Supplementary_Material

arae005_suppl_Supplementary_Figure_S1

arae005_suppl_Supplementary_Figure_S2

arae005_suppl_Supplementary_Figure_S3

## Data Availability

Analyses reported in this article can be reproduced using the data and R code provided by Gangoso et al. (2024).
