# Supplementary material for: A new trophic specialization buffers a top predator against climate-driven resource instability
Source: Behav Ecol. 2024 Jan 17;35(2):arae005. doi: 10.1093/beheco/arae005 (PMC10824164; doi:10.1093/beheco/arae005)
Supplement: arae005_suppl_Supplementary_Material [file arae005_suppl_supplementary_material.pdf]

Supplementary Information

Article: A new trophic specialization buffers a top predator against climate-driven resource instability (Gangoso et al.)

Table S1. Summary statistics of the Generalized Linear Mixed Model GLMM with negative binomial distributed errors assessing the effects of male morph (dark, pale) and colony (Caldera, North, Rapalobos) on the number of petrels consumed. Alternative models (in R notation) with their respective Akaike Information Criterion (AIC) values and the comparisons between models performed in the likelihood ratio tests are shown.

Parameter estimates (for mp1)			Likelihood Ratio Test				
Model term	Estimate	SE	Predictor	Test	χ^2	df	p-value
Intercept	-2.98	0.24					
male-dark	0.73	0.36	male morph	mp0, mp3	13.74	1	<0.001
				mp2, mp4	5.34	1	0.02
colony-North	5.27	0.23	colony	mp0, mp4	855.62	2	<0.001
colony-Rapalobos	1.60	0.23		mp2, mp3	847.22	2	<0.001
male-dark*colony-North	-0.32	0.39	male*colony	mp1, mp2	7.19	2	0.03
male-dark*colony-Rapalobos	-1.23	0.51					

Fitted models:
mp0: petrel ~ (1|year) #AIC = 3505.6
mp1: petrel ~ male*colony+(1|year)) #AIC = 2647.4
mp2: petrel ~ male+colony+(1|year) #AIC = 2650.6
mp3: petrel ~ male+(1|year) #AIC = 3493.8
mp4: petrel ~ colony+(1|year) #AIC = 2653.9

Table S2. Summary statistics of the GLMM (negative binomial) assessing the effects of male morph (dark, pale) on the number of petrels consumed only in the North colony. Alternative models (in R notation) with their respective Akaike Information Criterion (AIC) values and the comparisons between models performed in the likelihood ratio tests are shown.

Parameter estimates			Likelihood Ratio Test				
Model term	Estimate	SE	Predictor	Test	χ^2	df	p-value
Intercept	2.41	0.07					
male-dark	0.27	0.11	male morph	mp0, mp1	6.12	1	0.01

Fitted models:
mp0: petrel ~ (1|year) #AIC = 1663
mp1: petrel ~ male+(1|year)) #AIC = 1658.8

Table S3. Summary statistics of the GLMM model (Poisson distributed errors) assessing the effects of different variables affecting the daily number of preys delivered to nestlings. Alternative models are shown. Because only the model including the number of nestlings present and prey availability (m15) ranked first in terms of AIC (bolded), we only performed the likelihood ratio test for these 2 variables.

Parameter estimates (model ml18)			Likelihood Ratio Test				
Model term	Estimate	SE	Predictor	Test	χ^2	df	p-value
Intercept	2.42	0.60					
Number of nestlings present	0.14	0.03	nestlings	m8, m18	16.98	1	<0.001
male-pale	-0.03	0.07					
colony-Caldera	-0.060	0.10					
colony-North	-0.13	0.08					
colony-Rapalobos	0.09	0.09					
nestlings' age	-0.00	0.00					
prey availability	-0.03	0.01	prey	m4, m18	40.44	1	<0.001

Fitted models:
m0: prey ~ (1|nest) #AIC = 3283.9
m1: prey ~ nestlings + (1|nest) #AIC = 3270.3
m2: prey ~ nestlings + male + (1|nest) #AIC = 3272.2
m3: prey ~ nestlings + male + colony + (1|nest) #AIC = 3273.1
m4: prey ~ nestlings + male + colony + age + (1|nest) #AIC = 3274.9
m5: prey ~ male + (1|nest) #AIC = 3285.3
m6: prey ~ male + colony + (1|nest) #AIC = 3288.9
m7: prey ~ male + colony + age + (1|nest) #AIC = 3290.8
m8: prey ~ male + colony + age + u_wind + (1|nest) #AIC = 3251.4
m9: prey ~ colony + (1|nest) #AIC = 3286.9
m10: prey ~ colony + age + (1|nest) #AIC = 3288.9
m11: prey ~ colony + age + u_wind + (1|nest) #AIC = 3249.4
m12: prey ~ age + (1|nest) #AIC = 3285.9
m13: prey ~ age + u_wind + (1|nest) #AIC = 3245.7
m14: prey ~ u_wind + (1|nest) #AIC = 3245.8
m15: prey ~ nestlings+ u_wind + (1|nest) #**AIC = 3232.8**
m16: prey ~ nestlings+ colony + (1|nest) #AIC = 3271.1
m17: prey ~ nestlings+ age + (1|nest) #AIC = 3271.1
ml18: prey ~ nestlings + male + colony + age + u_wind + (1|nest) #AIC = 3283.9

Table S4. Summary statistics of the Linear Mixed Model assessing the relationship between the number of petrels in diet and the laying date. Alternative models and the comparisons performed in the likelihood ratio tests are shown.

Parameter estimates			Likelihood Ratio Test				
Model term	Estimate	SE	Predictor	Test	χ^2	df	p-value
Intercept	23.08	0.58					
number of petrels	-0.26	0.03	petrel	ml0, ml1	74.87	1	<0.001

Fitted models:
ml0: laying_date ~ (1|year) #AIC = 7487.05
ml1: laying_date ~ petrel+(1|year) #AIC = 7419.22

Table S5. Summary statistics of the Cumulative Link Mixed Model (CLMM) assessing the effects of petrels in diet and their interaction with food availability and the laying date on the number of fledglings raised. Alternative models and the comparisons performed in the likelihood ratio tests are shown.

Parameter estimates			Likelihood Ratio Test				
Model term	Estimate	SE	Predictor	Test	χ^2	df	p-value
Model fm4							
number of petrels	0.07	0.01	petrel	fm0, fm1	49.95	1	<0.001
				fm2, fm3	48.86	1	<0.001
prey availability	0.57	0.13	prey	fm0, fm3	12.11	1	<0.001
				fm1, fm2	11.01	1	<0.001
number of petrels*prey availability	-0.01	0.01	petrel*prey	fm2, fm4	3.02	1	0.08
Model fm6							
number of petrels	0.14	0.04	petrel	fm5, fm7	45.47	1	<0.001
mean annual productivity	2.20	0.18	productivity	fm0, fm7	40.44	1	<0.001
				fm1, fm5	35.96	1	<0.001
number of petrels*mean annual productivity	-0.05	0.02	petrel*productivity	fm5, fm6	4.60	1	0.03
Model fm8							
laying date	-0.09	0.01	laying date	fm0, fm8	101.18	1	<0.001

Fitted models:
fm0: fledglings ~ 1+(1|year) #AIC = 3302.05
fm1: fledglings ~ petrel + (1|year) #AIC = 3254.09
fm2: fledglings ~ petrel+prey + (1|year) #AIC = 3245.08
fm3: fledglings ~ prey + (1|year) #AIC = 3291.94
fm4: fledglings ~ petrel*prey + (1|year) #AIC = 3244.06
fm5: fledglings ~ petrel+prod + (1|year) #AIC = 3220.14
fm6: fledglings ~ petrel*prod + (1|year) #AIC = 3217.54
fm7: fledglings ~ prod + (1|year) #AIC =3263.61
fm8: fledglings ~ laying_date + (1|year) #AIC = 2194.95

Table S6. Summary statistics of the Linear Models (LM) assessing (a) changes in the mean number of petrels hunted across years, (b) the effects of the interaction between year and colony and (c) the effects of the interaction between the colony and the mean number of petrels in diet on the mean number of nests.

Parameter estimates			Significance test			
Model	Estimate	SE	Predictor	F	df	p-value
(a) mean number of petrels ~ year						
Intercept	-365.57	58.30				
year	0.18	0.03	year	39.80	1	<0.001
(b) mean number of nests ~ year * colony						
Intercept	-0.50	21.90				
year	0.00	0.01	year	67.23	1	<0.001
colony-North	-249.71	31.00	colony	125.05	2	<0.001
colony-Rapalobos	-50.67	31.00				
year*colony-North	0.12	0.02	year*colony	35.88	2	<0.001
year*colony-Rapalobos	0.03	0.02				
(c) mean number of nests ~ mean number of petrels * colony						
Intercept	3.68	0.16				
petrel.mean	-0.02	0.07	petrel	24.93	1	<0.001
colony-North	-2.30	0.23	colony	72.14	2	<0.001
colony-Rapalobos	-0.32	0.23				
petrel.mean *colony-North	0.56	0.09	petrel*colony	19.37	2	<0.001
petrel.mean *colony-Rapalobos	0.09	0.09				
