# Supplementary figures and images for: A new trophic specialization buffers a top predator against climate-driven resource instability
Source: Behav Ecol. 2024 Jan 17;35(2):arae005. doi: 10.1093/beheco/arae005 (PMC10824164; doi:10.1093/beheco/arae005)

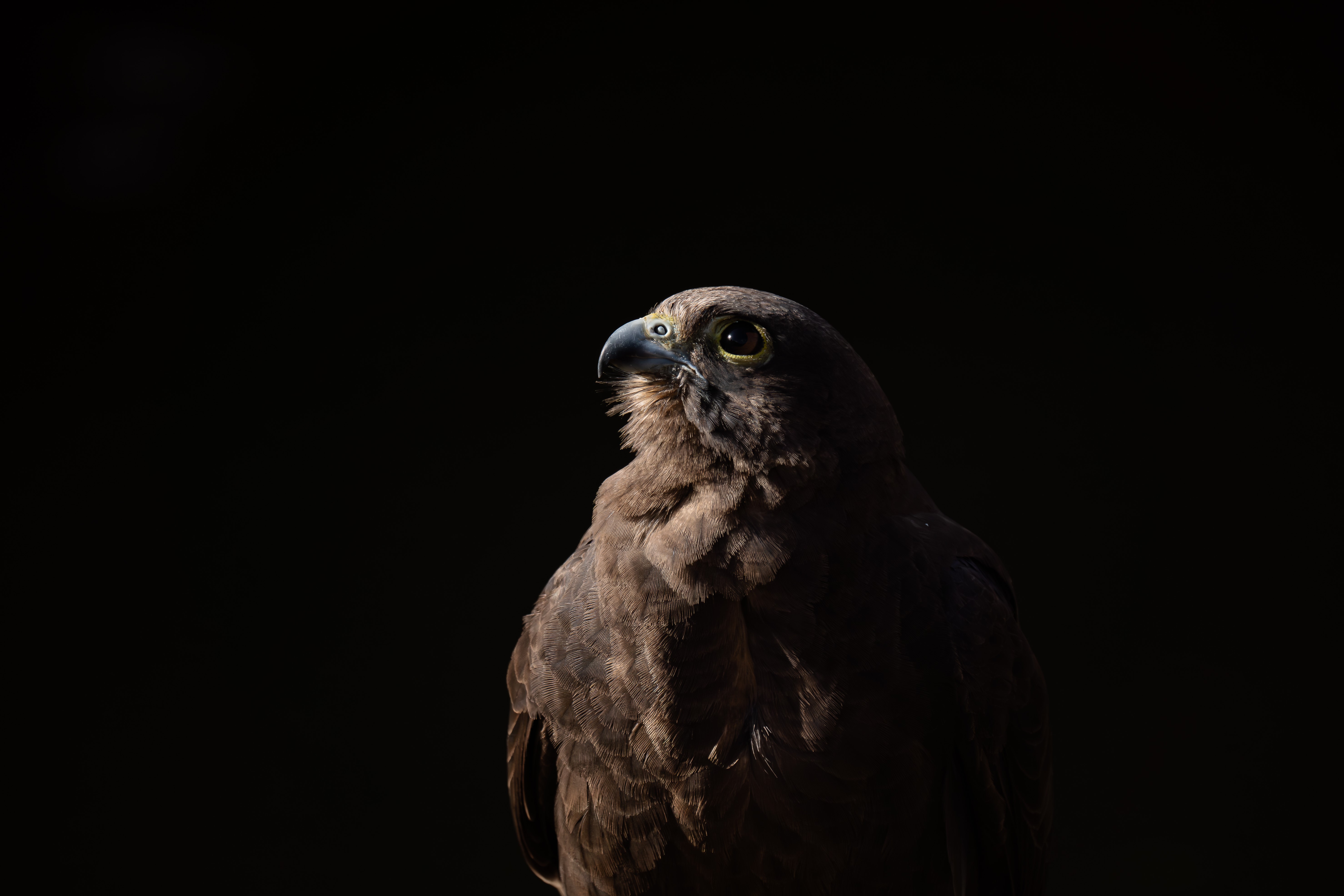

Supplement: arae005_suppl_Supplementary_Figure_S1 [file arae005_suppl_supplementary_figure_s1.jpeg]

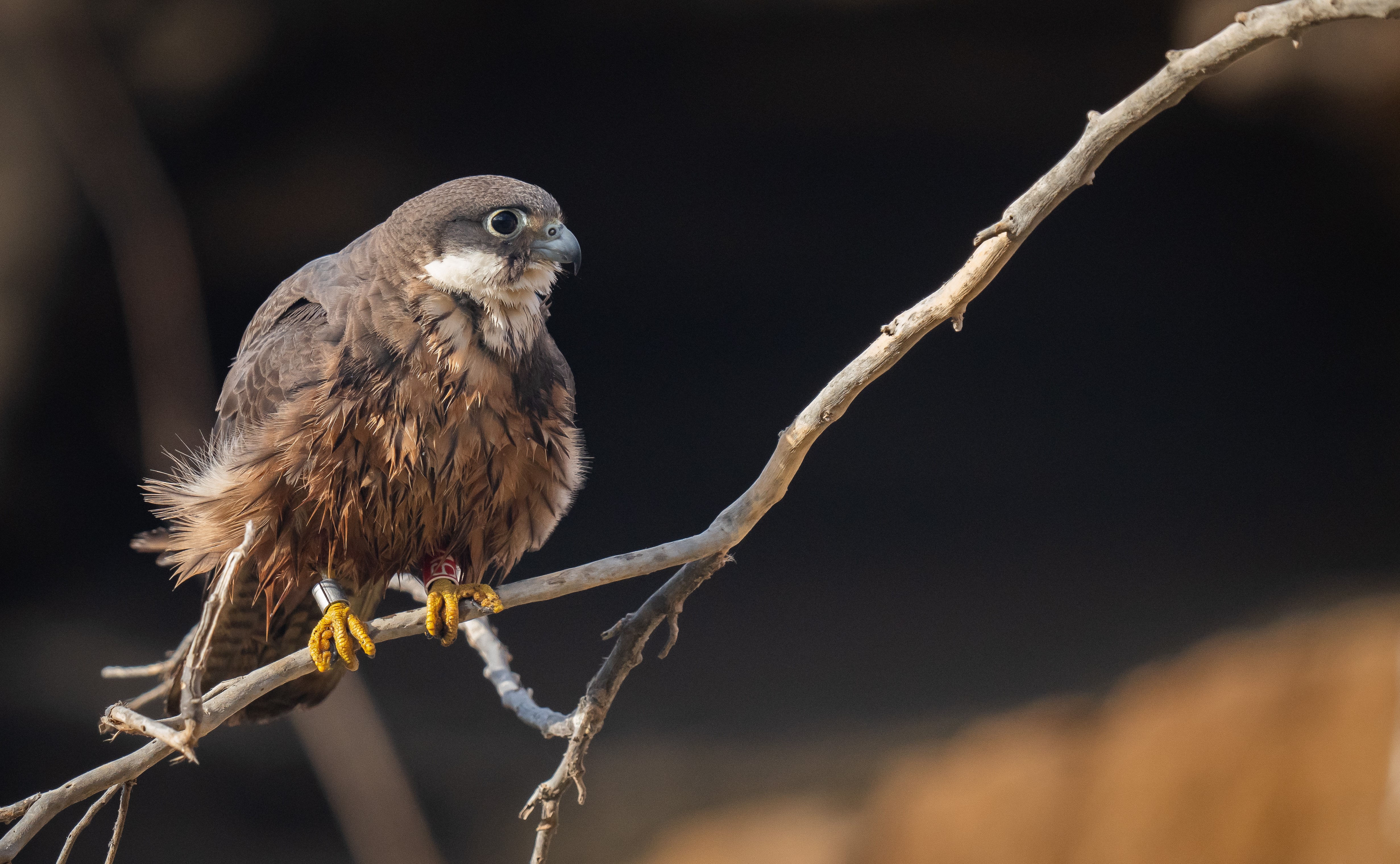

Supplement: arae005_suppl_Supplementary_Figure_S2 [file arae005_suppl_supplementary_figure_s2.jpeg]

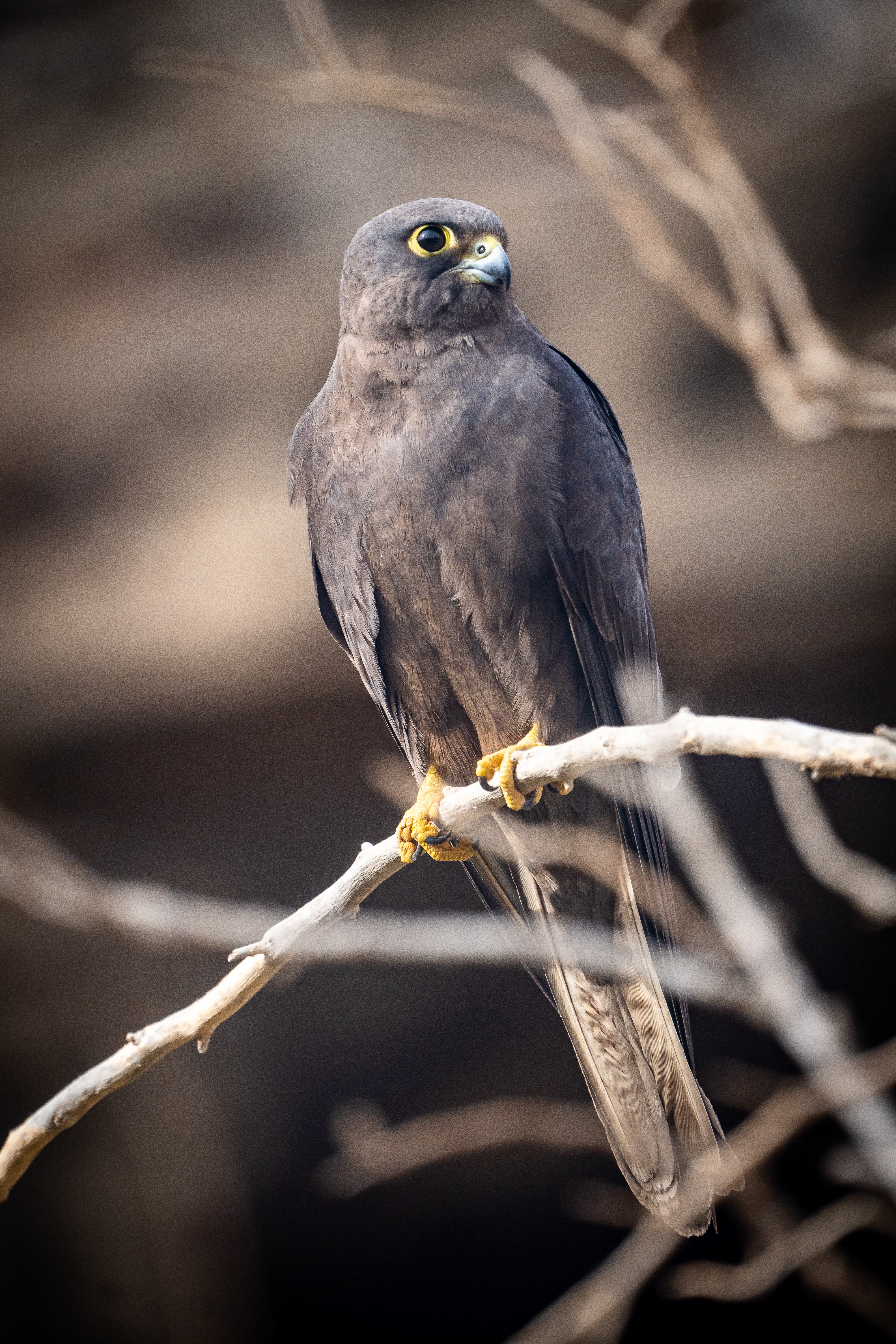

Supplement: arae005_suppl_Supplementary_Figure_S3 [file arae005_suppl_supplementary_figure_s3.jpeg]
